# The retinol-metabolizing enzyme DHRS3 coordinates antigen presentation, endothelial stability, and cholesterol metabolism to suppress hepatocellular carcinoma progression

**DOI:** 10.1016/j.jbc.2026.113168

**Published:** 2026-05-15

**Authors:** Hongtao Hu, Lidan Zhang, Jiawen Gu, Jialun Zhang, Ying Lu, Kai Zhang, Chenhao Shi, Hang Gao, Siyue Lou, Haixin Qin, Huajun Zhao

**Affiliations:** 1 School of Pharmaceutical Sciences, Zhejiang Chinese Medical University, Hangzhou, Zhejiang, China; 2 Academy of Chinese Medical Sciences, Zhejiang Chinese Medical University, Hangzhou, Zhejiang, China

**Keywords:** hepatocellular carcinoma, DHRS3, tumor microenvironment, retinoic acid signaling, cholesterol metabolism, machine learning

## Abstract

Hepatocellular carcinoma (HCC) remains a therapeutic challenge due to tumor microenvironment heterogeneity and metabolic reprogramming. Short-chain dehydrogenase/reductase 3, DHRS3, a retinol-metabolizing enzyme, exhibits paradoxical roles across cancers, but its specific function in HCC is poorly understood. This study integrated pan*-*cancer data from The Cancer Genome Atlas and genotype-tissue expression, conducted single-cell and spatial transcriptomic analyses, and carried out *in vitro* assays and *in vivo* xenograft models for validation. A machine learning framework was used to develop the DHRS3-cholesterol metabolic prognostic signature (DCMPS) by combining DHRS3 expression with cholesterol-related genes. DHRS3 was upregulated in HCC and associated with favorable prognosis. It enhanced antitumor immunity by promoting major histocompatibility complex class I/II antigen presentation and inhibited metastasis *via* SEMA4A signaling and endothelial-mesenchymal transition suppression. Conversely, DHRS3 drove proliferation through cholesterol metabolism, reversible by atorvastatin (ATO). ATO synergized with all-trans retinoic acid to suppress tumor growth and enhance immune cell infiltration. The DCMPS model outperformed Tumor Node Metastasis staging in prognostic prediction. DHRS3 acts as a dual-functional immune-metabolic regulator in HCC. The DCMPS signature serves as a robust prognostic biomarker and may guide the application of all-trans retinoic acid-ATO combination therapy, offering a rationale for a novel precision medicine strategy in HCC management.

Primary liver cancer is currently one of the most prevalent malignancies worldwide. According to statistics from the American Cancer Society (1), hepatocellular carcinoma (HCC) accounts for approximately 75 to 85% of primary liver cancer cases, emerging as the sixth most commonly diagnosed cancer and the third leading cause of cancer-related deaths globally. As a highly heterogeneous disease, HCC exhibits a poor prognosis, with a median survival time of 6 to 20 months after diagnosis and a 5-year survival rate below 20%, showing closely aligned incidence and mortality rates (2, 3).

Despite advances guided by evidence-based clinical guidelines, therapeutic outcomes for HCC remain limited (4). Current clinical strategies encompass radiotherapy, chemotherapy, hepatic resection, orthotopic liver transplantation, local ablation, and intra-arterial therapies (5). Among these, hepatic resection and liver transplantation demonstrate relative effective, achieving 10-year recurrence-free survival rates of 22 to 25% and 50 to 70%, respectively (6). However, these interventions are predominantly applicable to patients in early-stage HCC, whereas most patients present with advanced, inoperable disease at diagnosis (7, 8). Furthermore, systemic therapy serves as the primary approach for advanced HCC, employing first-line agents (*e.g.*, sorafenib, lenvatinib) and second-line options (*e.g.*, regorafenib, cabozantinib, ramucirumab) (9, 10). Regrettably, acquired therapeutic resistance undermines targeted interventions—exemplified by sorafenib’s efficacy, which only benefits ∼30% of patients, with resistance typically emerging within 6 months (11). Similarly, HCC exhibits pronounced resistance to conventional chemotherapeutics like 5-fluorouracil and doxorubicin (12). Collectively, these challenges underscore the urgent need for novel therapeutic paradigms in HCC management.

Short-chain dehydrogenase/reductase 3 (DHRS3), a core member of the short-chain dehydrogenase/reductase family, dynamically regulates vitamin A metabolism by catalyzing the reversible oxidation of all-trans retinol to all-trans retinaldehyde (13), thereby fine-tuning the biosynthesis and homeostasis of all-trans retinoic acid (ATRA) (14, 15, 16). ATRA, a bioactive metabolite of vitamin A, exhibits broad therapeutic potential in cancer, such as suppressing HCC progression *via* inhibition of Pin1 activity (17, 18) or modulation of the OTUD7B pathway (19). Notably, DHRS3 expression is feedback-enhanced by ATRA treatment (20, 21), underscoring its dual role as both an effector and regulatory node in the retinol metabolic axis. However, DHRS3 exhibits context-dependent dual roles in cancer. It functions as a tumor suppressor in gastric cancer, where it is frequently silenced by promoter hypermethylation (22). In contrast, DHRS3 demonstrates a functional duality in triple-negative breast cancer, promoting tumor cell proliferation (23) yet conversely suppressing metastatic potential (24). Similarly, in radiation-induced thyroid carcinoma (THCA), DHRS3 is upregulated (25) and its elevated expression is associated with inhibition of metastasis (26). This cross-cancer mechanistic dichotomy highlights the context-dependent reprogramming of DHRS3 functions by tumor microenvironments. It is particularly worth noting that DHRS3 exhibits tissue-specific high expression in human liver, testes, and small intestine (27) and has been identified as an allelic expression imbalance gene in HCC (28). Nevertheless, its precise role and molecular mechanisms in HCC remain enigmatic. Dysregulated ATRA signaling is closely associated with HCC recurrence (29, 30) and chemoresistance (31, 32). As a central regulator of this pathway, DHRS3 may influence HCC progression through genetic variation, metabolic reprogramming, or modulation of cancer stem cell differentiation. Elucidating the HCC-specific regulatory network of DHRS3 will not only reconcile its paradoxical oncogenic-protective duality observed in pan-cancer but also pave the way for novel therapeutic strategies targeting vitamin A metabolism to address current challenges in HCC treatment, including drug resistance and the scarcity of targets.

This study reveals the dual functionality of DHRS3 in HCC, demonstrating its role in enhancing antitumor immunity through upregulation of major histocompatibility complex class I/II (MHC-I/II) antigen presentation and inhibition of endothelial-mesenchymal transition (EndMT), thereby remodeling the immune microenvironment. Conversely, DHRS3 promotes tumor proliferation *via* a cholesterol metabolism pathway, an effect that is effectively reversible through atorvastatin (ATO) treatment. Notably, ATO synergizes with ATRA to suppress tumor growth and enhance immune responses *in vivo*. Based on these mechanisms, we developed a machine learning–based prognostic signature (DHRS3-cholesterol metabolic prognostic signature [DCMPS]) integrating DHRS3 and cholesterol metabolism genes. DCMPS outperformed traditional Tumor Node Metastasis (TNM) staging and tumor mutational burden (TMB) in cross-cohort validation and effectively stratified patients for ATRA-ATO combination therapy. These findings establish DHRS3 as a key regulator of immune-metabolic crosstalk in HCC, providing a novel biomarker for prognosis and a actionable target for combinatorial therapy.

## Results

### Pan*-*cancer analysis of DHRS3 expression and its prognostic relevance

**Figure 1:**
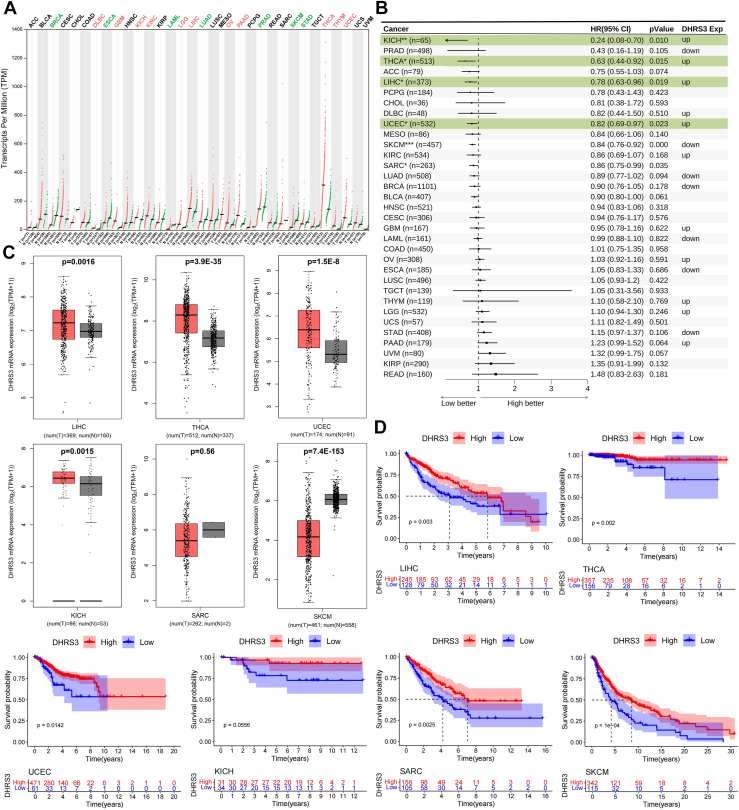
**Pan-cancer analysis of DHRS3 expression and its prognostic relevance.***A,* DHRS3 mRNA expression levels in tumor tissues (*red*) *versus* normal tissues (*green*) across 33 cancer types from TCGA and GTEx databases. Cancer types are color-coded to indicate differential DHRS3 expression in tumors (*red*: significant upregulation; *green*: significant downregulation; *black*: no significant change). *B,* univariate COX regression analysis of DHRS3 expression’s effect on prognosis in 33 cancers. *Green bars* denote cancers where high DHRS3 expression in tumors correlates with favorable prognosis. *C,* DHRS3 expression levels in tumor tissues *versus* normal tissues for six cancers (LIHC, THCA, UCEC, KICH, SARC, and SKCM) from TCGA and GTEx databases. *D,* Kaplan–Meier survival analysis of six cancers (LIHC, THCA, UCEC, KICH, SARC, and SKCM). High-DHRS3 patients exhibited significantly prolonged overall survival compared with low-DHRS3 patients. One-way ANOVA (*A* and *C*) and Cox proportional hazard regression models (*B*) or log-rank tests (*D*) were used for statistical analysis. DHRS3, short-chain dehydrogenase/reductase 3; GTEx, genotype-tissue expression; SARC, sarcoma; SKCM, skin cutaneous melanoma; TCGA, The Cancer Genome Atlas; THCA, thyroid carcinoma; UCEC, uterine corpus endometrial carcinoma; KICH, kidney chromophobe; LIHC, liver hepatocellular carcinoma.

To explore the pan-cancer role of DHRS3, we analyzed its mRNA expression across 33 cancer types using The Cancer Genome Atlas (TCGA) and genotype-tissue expression (GTEx) data *via* GEPIA (33) DHRS3 was significantly upregulated in 11 tumor tissues (*e.g.*, liver hepatocellular carcinoma [LIHC], kidney chromophobe [KICH], THCA) and downregulated in seven tumor tissues (*e.g.*, skin cutaneous melanoma [SKCM], sarcoma [SARC]) compared with normal tissues (Fig. 1*A*). Univariate Cox regression revealed that low DHRS3 expression lead to poor prognosis in six cancer types (LIHC, KICH, THCA, SKCM, uterine corpus endometrial carcinoma [UCEC], SARC; hazard ratio (HR) < 1, *p* < 0.05) (Fig. 1*B*). Notably, DHRS3 expression was paradoxically elevated in tumor tissues of LIHC, THCA, UCEC, and KICH (*versus* normal tissues) but strongly associated with favorable survival (Fig. 1, *C* and *D*). For LIHC, median overall survival (OS) was significantly longer in DHRS3-high patients than DHRS3-low patients (Fig. 1*D*). Mendelian randomization (Using DMRdb (34)) further supported the tumor-suppressive role of DHRS3, showing that genetically predicted DHRS3 overexpression reduced connective and soft tissue malignancy risk (Fig. S1). These findings highlight DHRS3 as a prognostic biomarker with cancer-type specific expression patterns and survival associations, particularly revealing its paradoxical overexpression-prognosis correlation in LIHC that warrants mechanistic investigation.

### Feedback-driven DHRS3 expression in HCC cells activates antitumor immunity

**Figure 2:**
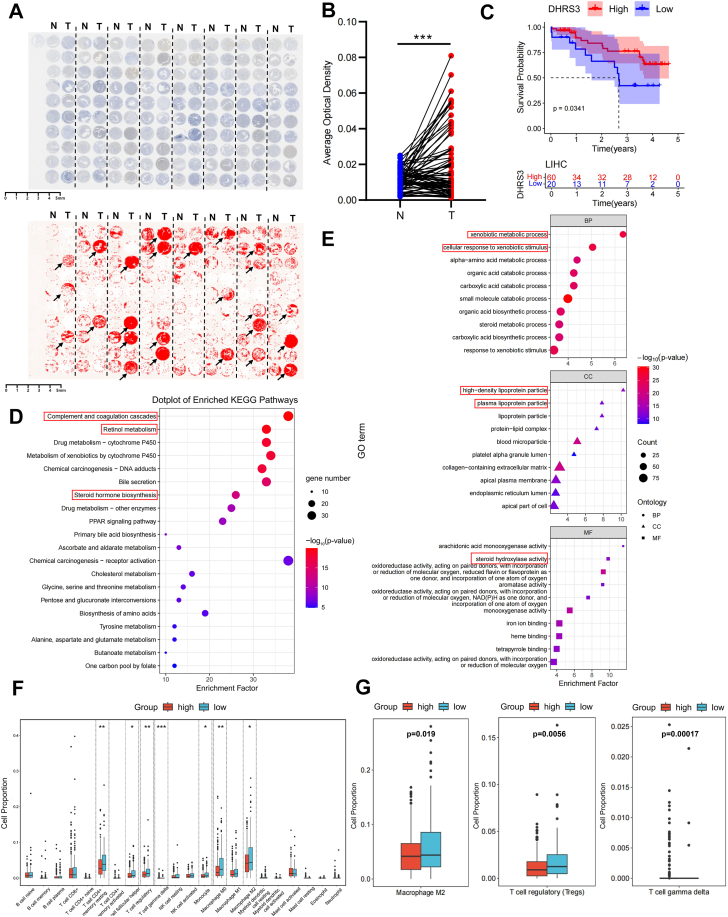
**Feedback-driven DHRS3 expression in hepatocellular carcinoma activates antitumor immunity.***A,* IHC detection of DHRS3 protein expression in tissue microarrays of 80 paired clinical HCC and adjacent nontumor samples (N: adjacent nontumor tissue; T, tumor tissue; *top*, representative IHC images; *bottom*, DHRS3 expression levels). *B,* statistical analysis of DHRS3 protein expression in tumor tissues (T) *versus* adjacent nontumor tissues (N). *C,* survival analysis based on DHRS3 expression, showing prolonged overall survival in HCC patients with high DHRS3 expression compared with those with low expression. *D* and *E,* KEGG enrichment analysis (*D*) and GO enrichment analysis (*E*) of differentially expressed genes (DEGs, *p* < 0.01, |log2FC| >1) between DHRS3 high- and low-expression HCC samples from the TCGA database. *F,* immune cell infiltration levels were evaluated by the CIBERSORT_abs algorithm in HCC tissues with high *versus* low DHRS3 expression. *G,* proportional changes in M2 macrophages, regulatory T cells, and γδ T cells in DHRS3 high-expression HCC tissues. Pair *t-*tests (*B*) and log-rank tests (*C*) or one-way ANOVA (*G*) were used for statistical analysis. ∗∗∗*p* < 0.001. KEGG, Kyoto Encyclopedia of Genes and Genomes; DHRS3, short-chain dehydrogenase/reductase 3; HCC, hepatocellular carcinoma; immunohistochemistry; GO, gene ontology; HCC, hepatocellular carcinoma; TCGA, The Cancer Genome Atlas.

To investigate the role of DHRS3 in HCC, we performed immunohistochemistry (IHC) on 80 paired HCC and adjacent nontumor tissue samples (Fig. 2*A*). Consistent with database-derived mRNA profiles, DHRS3 protein levels were significantly upregulated in tumor tissues compared to matched normal tissues (Fig. 2*B* and S2*A*), and high DHRS3 expression associated with improved patient survival (Fig. 2*C*).

The analysis of TCGA-LIHC data revealed elevated DHRS3 expression in male patients and elder individuals (Fig. S2, *B* and *C*), independent of TNM stage (Fig. S2*D*). These findings were validated in our cohort, where DHRS3 expression positively correlated with age (Fig. S2, *E*–*H*). Given the age-dependent accumulation of somatic mutations, we assessed associations between DHRS3 expression and TMB or microsatellite instability (MSI). DHRS3 expression showed a strongly positive correlated with TMB (Fig. S2, *I*–*J*), consistent with prior reports of DHRS3 upregulation in irradiation-induced thyroid cancers with copy number alternations (25), supporting a feedback-driven mechanism that promotes DHRS3 expression during malignant progression.

Differential gene expression analysis (limma, *p* < 0.01, |log2FC|>1) between DHRS3-high and DHRS3-low HCC groups identified enriched Kyoto Encyclopedia of Genes and Genomes pathways including complement/coagulation cascades, retinol metabolism, and steroid hormone biosynthesis (Fig. 2*D*). Gene ontology analysis highlighted xenobiotic metabolism, cellular responses to xenobiotic stimuli (biological processes), arachidonic acid/steroid hydroxylase activities (molecular functions), and high-density lipoprotein particles (cellular components) (Fig. 2*E*). These enrichment findings highlight that DHRS3 not only serves as a pivotal enzyme in retinol metabolism but also implicates its dual regulatory roles in immune modulation and cholesterol-associated metabolic pathways.

CIBERSORT_abs-based immune cell infiltration analysis demonstrated significant differences of monocytes, M0/M2 macrophages, resting CD4+ memory T cells, follicular helper T cells, gamma-delta T cells, and regulatory T cells between DHRS3-high and DHRS3-low groups (Fig. 2*F*). In the DHRS3-high group, immunosuppressive M2 macrophages and regulatory T cells were markedly reduced, while γδ T cells with potent tumor-killing capacity, increased significantly (Fig. 2*G*). These results indicate that feedback-driven DHRS3 expression in HCC cells enhances antitumor immunity, reconciling its paradoxical relationship between tumor expression and favorable prognosis.

### DHRS3+ cells demonstrate enhanced intercellular communication with reduced abundance at the invasive fronts

**Figure 3:**
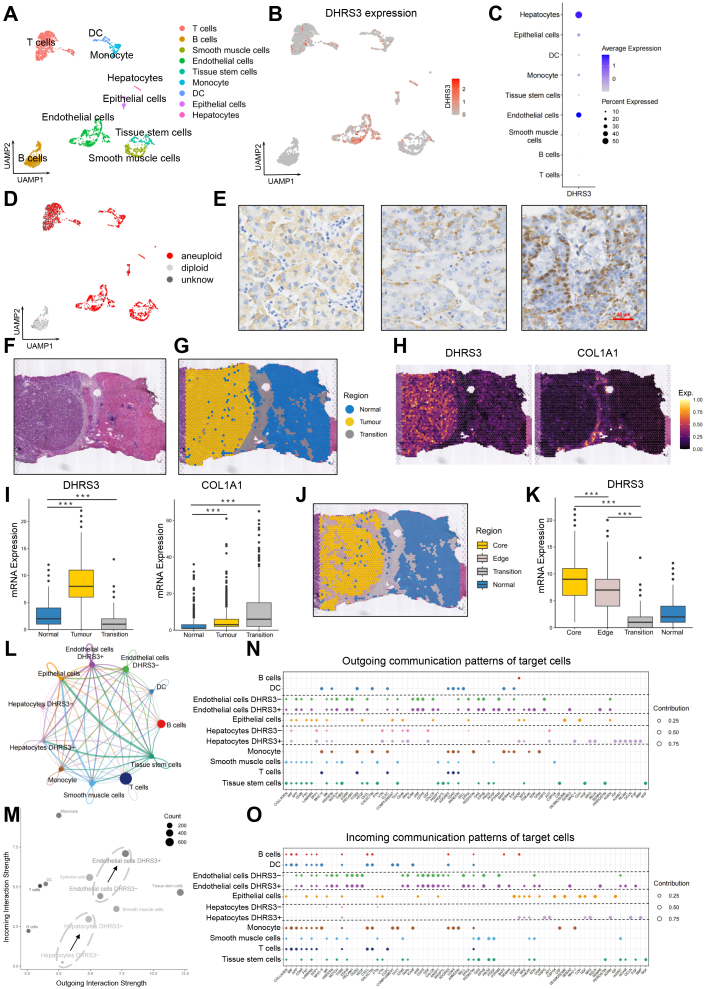
**DHRS3+ cells demonstrate enhanced intercellular communication with reduced abundance at the invasive fronts.***A,* UMAP projection of major cell clusters in HCC tissues from 19 patients (GSE125449). *B,* DHRS3 expression heatmap across cell types (*red*: high expression; *gray*: low). *C,* bubble plot illustrating DHRS3 expression levels and proportions in different cell types. *D,* CopyKAT-inferred copy number variations in HCC cells. *E,* IHC validation of cell type–specific DHRS3 expression in human HCC. *F,* H&E staining of HCC invasive front. *G,* spatial demarcation of tumor, normal, and transitional zones. *H,* spatial expression patterns of DHRS3 and invasion marker COL1A1. *I,* quantification of DHRS3 and COL1A1 expression across zones. *J,* subregional analysis of DHRS3 expression from tumor core to margin. *K,* statistical comparison of DHRS3 expression between core and marginal regions. *L,* CellChat-derived interaction network among cell types (line width: interaction strength). *M,* comparison of incoming/outgoing signal intensities between DHRS3+ and DHRS3− hepatocytes/endothelial cells. *N* and *O,* dot plots showing enriched incoming (*N*) and outgoing (*O*) ligand-receptor pairs across cell types. One-way ANOVA (*I* and *K*) was used for statistical analysis. ∗∗∗*p* < 0.001. UMAP, uniform manifold approximation and projection; DHRS3, short-chain dehydrogenase/reductase 3; HCC, hepatocellular carcinoma; IHC, immunohistochemistry.

To elucidate the mechanism by which DHRS3 activates antitumor immunity, we analyzed single-cell transcriptomic data from 19 HCC patients (GSE125449) in the GEO database. Uniform manifold approximation and projection dimensionality reduction and clustering found major cell populations (Fig. 3*A*). SingleR annotation demonstrated that DHRS3 was predominantly expressed in hepatocytes and endothelial cells (Fig. 3, *B* and *C*). CopyKAT analysis showed widespread copy number variations across cell types, confirming malignant transformation in most cells (Fig. 3*D*). IHC validation of clinical tissue microarrays corroborated cell type–specific DHRS3 expression in HCC tissues (Fig. 3*E*).

We performed secondary analysis on previously published spatial transcriptomic data from HCC invasive fronts (35). Spatial coordinates and gene expression profiles were integrated to cluster sample spots, enabling partitioning of tumor, adjacent normal, and transition zones (Fig. 3, *F* and *G*). Consistent with prior findings, DHRS3 exhibited marked upregulation in tumor regions. Intriguingly, its expression in transition zones was significantly reduced compared with normal tissues (Fig. 3, *H* and *I*). Further spatial stratification within tumor regions revealed progressively decreasing DHRS3 expression from the tumor core to peripheral margins, demonstrating a radial expression gradient that inversely correlated with distance from the core (Fig. 3, *J* and *K*). This spatially resolved expression pattern implies a specialized role for DHRS3 in regulating tumor cell metastasis and intercellular crosstalk. Low-DHRS3–expressing cells at the tumor periphery may develop enhanced metastatic capacity and immune evasion, forming a protective barrier that shields the tumor bulk while facilitating metastatic spread.

CellChat analysis quantified intercellular communication strength between DHRS3± hepatocytes/endothelial cells and other cell types. DHRS3+ cells exhibited significantly enhanced input/output interaction intensities compared with DHRS3− counterparts (Fig. 3, *L* and *M*). Detailed ligand-receptor pair analysis revealed substantial signal loss in DHRS3− cells across multiple pathways (Fig. 3, *N* and O). These results collectively indicate that DHRS3+ cells possess enhanced intercellular communication networks, while their depletion at invasive fronts may create microenvironments for tumor progression.

### DHRS3 promotes SEMA4A signaling in endothelial cells and inhibits EndMT

**Figure 4:**
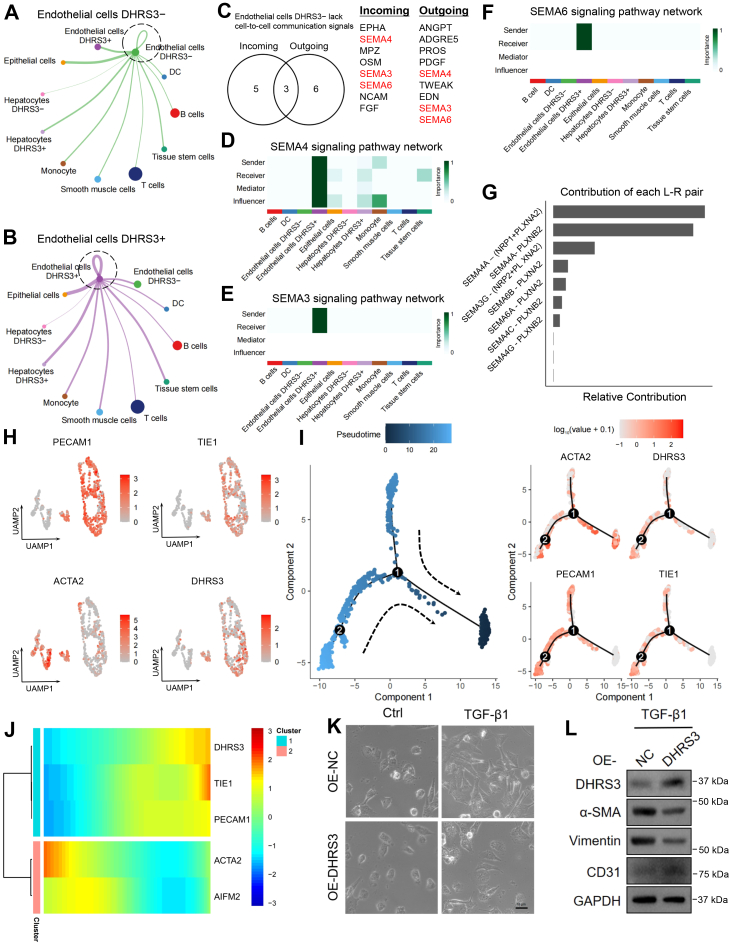
**DHRS3 promotes SEMA4A signaling in endothelial cells and inhibits EndMT.***A* and *B,* CellChat analysis of Interaction strength between DHRS3^−^ (*A*) or DHRS3+ (*B*) endothelial cells and other cell types (line width: interaction strength). *C,Venn diagram* of differentially enriched input/output signals between DHRS3+ and DHRS3− endothelial cells. *D* and *F,* CellChat analysis of SEMA3 (D), SEMA4 (*E*), and SEMA6 (*F*) pathways signaling flow patterns in HCC tissues. *G,* CellChat analysis of relative contributions of specific ligand (L)-receptor (R) pairs in SEMA3/4/6 signaling. *H,* UMAP analysis of endothelial and mesenchymal cells colored by EndMT-related markers and DHRS3 expression. *I,* monocle2-based pseudotime trajectory analysis of EndMT-associated cells. *J,* dynamic expression heatmap of EndMT-related genes and DHRS3 during transition. *K,* phase-contrast images showing the effects of DHRS3 overexpression on TGF-β1–induced EndMT morphology in SK-HEP-1 cells. Cells were treated with 10 ng/ml TGF-β1 for 48 h to induce EndMT. *L,* Western blot analysis of EndMT-related proteins in SK-HEP-1 cells following DHRS3 overexpression and TGF-β1 treatment. UMAP, uniform manifold approximation and projection; DHRS3, short-chain dehydrogenase/reductase; HCC, hepatocellular carcinoma; endothelial-mesenchymal transition.

To investigate the enhanced intercellular communication mediated by DHRS3+ endothelial cells, we first analyzed the interaction strength between DHRS3± endothelial cells and other cell types. Results revealed significantly enhanced autocrine signaling in DHRS3+ endothelial cells (Fig. 4, *A* and *B*). Differential analysis of input and output signals between DHRS3+ and DHRS3− endothelial cells demonstrated upregulated SEMA3, SEMA4, and SEMA6 signaling pathways in DHRS3+ endothelial cells (Fig. 4*C*). Notably, DHRS3+ endothelial cells served as both primary senders and receivers in these three signaling pathways (Fig. 4, *D*–*F*). Further analysis of specific ligand-receptor pairs within these pathways identified SEMA4A-related signaling as the predominant contributor (Fig. 4*G*). SEMA4A, a known activator of T cell–mediated immunity (36), has been shown to inhibit vascular endothelial growth factor-induced endothelial cell migration *in vitro* and angiogenesis *in vivo* (37). PCR analysis confirmed that DHRS3 overexpression increased SEMA4A expression level in hepatic endothelial SK-HEP-1 cells (Fig. S3*A*), indicating the role of DHRS3 in migration regulation. Scratch wound healing assays demonstrated that DHRS3 overexpression significantly inhibited hepatic endothelial cell migration (Fig. S3*B*).

EndMT enhances endothelial cell migratory capacity through altering their phenotype and function, which is a critical driver in tumor metastasis. Previous studies revealed that Lats1/2-mutant cells arrested in an intermediate state during fibroblast differentiation exhibit upregulated Yap target gene Dhrs3 (38). Combined with spatial transcriptomic findings showing lower DHRS3 expression at invasive fronts, these observations suggest DHRS3-mediated regulation of EndMT to block metastasis. Reclustering of annotated endothelial (marked by PECAM1/CD31 and TIE1) and mesenchymal cells (ACTA2/α-SMA) (39) identified a transitional cell population coexpressing both markers (Fig. 4*H*). Pseudotime trajectory analysis demonstrated progressive DHRS3 downregulation alongside increasing expression of mesenchymal markers ACTA2 and AIFM2 (Fig. 4, *I* and *J*). Functional validation confirmed that DHRS3 overexpression suppressed TGF-β–induced EndMT, evidenced by reduced α-SMA and Vimentin expression alongside increased CD31 levels (Fig. 4, *K* and *L*). Collectively, these findings establish that DHRS3+ endothelial cells enhance SEMA4A-mediated intercell communication while inhibiting EndMT-associated migration.

### DHRS3 enhances MHC molecule expression in HCC cells to augment immunogenicity

**Figure 5:**
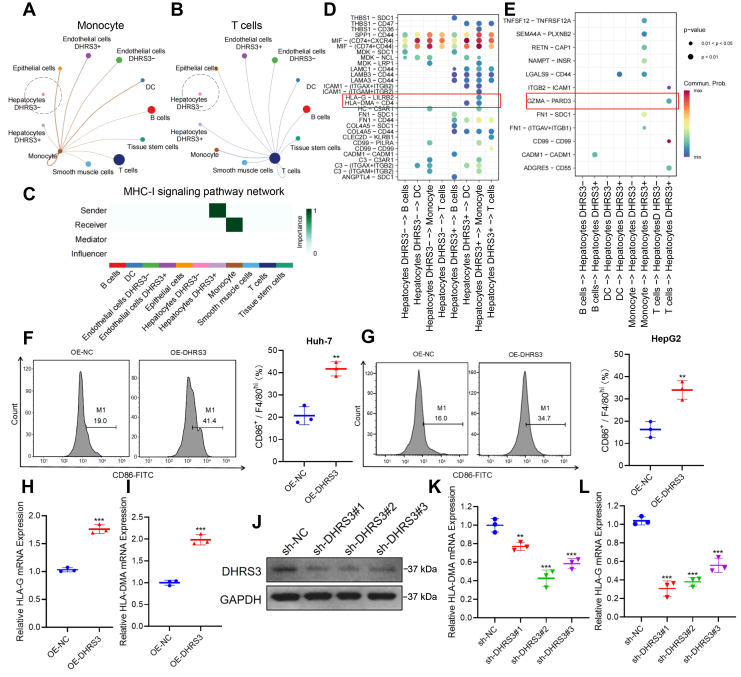
**DHRS3 promotes MHC molecule expression in HCC cells to enhance immunogenicity.***A* and *B,* CellChat analysis of Interaction strength between monocytes (A) or T cells (B) and HCC subpopulations (line width: interaction strength). DHRS3^−^ HCC cells exhibit attenuated communication with immune cells. *C,* CellChat analysis of MHC class I pathways Signaling flow patterns in HCC tissues. *D* and *E,dot plots* showing ligand-receptor pair deficits in DHRS3^−^ HCC cells for HLA-G, HLA-DMA (D), and GZMA (E) signaling. *F* and *G,* flow cytometry quantification of M1-polarized macrophages (CD86+/F4/80+) following coculture with DHRS3-overexpressing Huh-7 (F) or HepG2 (G) cells (n = 3). *H* and *I,* RT-qPCR analysis of HLA-G (H) and HLA-DMA (I) mRNA levels in DHRS3-overexpressing Huh-7 cells (n = 3). *J,* Western blot analysis of DHRS3 expression in shRNA-treated Huh-7 cells. *K* and *L,* RT-qPCR analysis of HLA-G (K) and HLA-DMA (L) mRNA levels in DHRS3-knockdown Huh-7 cells (n = 3). All experiments were performed with n independent biological replicates. The data are presented as mean ± SD values. One-way ANOVA was used for statistical analysis. ∗∗*p* < 0.01, ∗∗∗*p* < 0.001. GZMA, granzyme A; RT-qPCR, reverse transcription and quantitative real-time PCR; DHRS3, short-chain dehydrogenase/reductase; HCC, hepatocellular carcinoma.

To elucidate the mechanisms underlying the DHRS3+ HCC cells enhancing intercellular communication, comparative analysis revealed diminished interactions between DHRS3− HCC cells and critical tumor-associated immune populations. And this analysis showed reduced communication with T cells and monocytes in DHRS3− cells (Fig. 5, *A* and *B*). Pathway dissection identified a marked deficiency in MHC class I signaling within DHRS3− hepatocyte cells (Fig. 5*C*), a critical pathway for immune recognition of malignant cells (40). Notably, HLA-G–associated signaling and MHC class II molecules such as HLA-DMA (41) were significantly downregulated in DHRS3− cells (Fig. 5*D*). Furthermore, expression of granzyme A, a pivotal mediator of T cell–mediated tumor cytotoxicity (42), was substantially suppressed in these cells (Fig. 5*E*).

Functional validation was performed by coculturing phorbol 12-myristate 13-acetate-induced THP-1 macrophages with DHRS3-overexpressing Huh-7 or HepG2 cells. Flow cytometric analysis demonstrated enhanced M1 polarization of macrophages in the coculture system (Fig. 5, *F* and *G*), indicative of heightened antitumor immune activation. To validate these findings, DHRS3 overexpression in Huh-7 cells elevated expression of these immunoregulatory molecules (Fig. 5, *H* and *I*). DHRS3-knockdown Huh-7 cells were generated using shRNA, with efficacy confirmed by Western blotting (Fig. 5*J*). Conversely, DHRS3 knockdown specifically lowered HLA-G and HLA-DMA mRNA levels (Fig. 5, *K* and *L*). These findings collectively establish that DHRS3 potentiates HCC immunogenicity through coordinated upregulation of MHC class I/II molecules, thereby promoting immune-mediated tumor surveillance.

### DHRS3 modulates cholesterol metabolism to promote hepatocellular carcinoma progression

**Figure 6:**
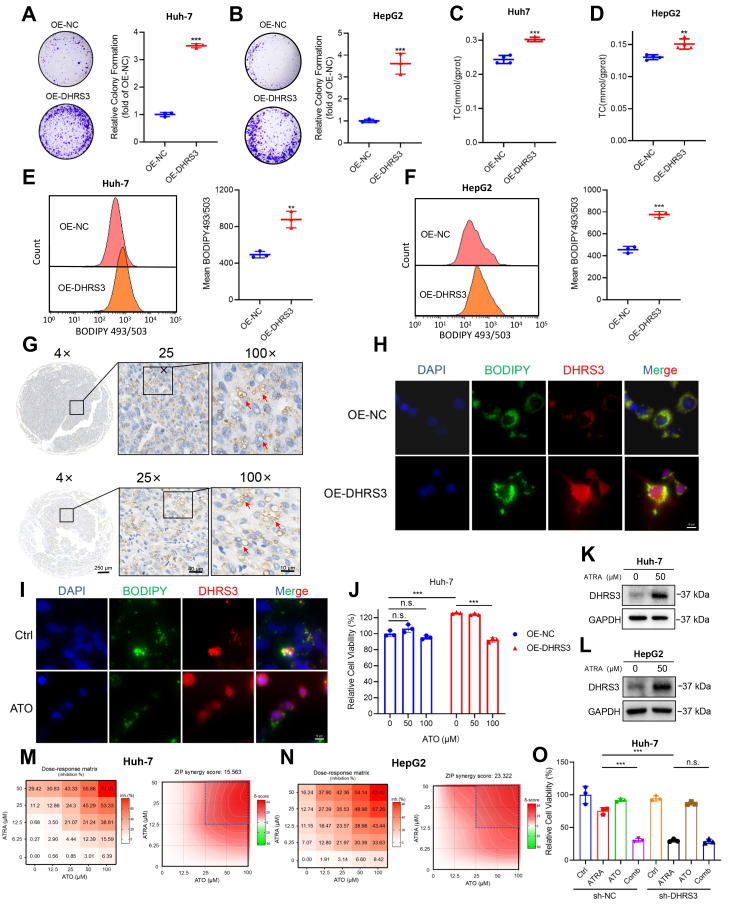
**DHRS3 regulates cholesterol metabolism to drive HCC proliferation.***A* and *B,* colony formation assay detected the effect of DHRS3 overexpression on the proliferative capacity of Huh-7 (A) or HepG2 (B) cells (n = 3). *C* and *D,* kit assay detected the effect of DHRS3 overexpression on Total cholesterol (TC) content in Huh-7 (C) or HepG2 (D) cells (n = 4). *E* and *F,* BODIPY staining was used to detect the effect of DHRS3 overexpression on lipid droplet content in Huh-7 (E) or HepG2 (F) cells (n = 3). *G,* IHC detected DHRS3 expression in lipid droplet areas of HCC cells. *H,* DHRS3 immunofluorescence and BODIPY staining were performed in DHRS3-overexpressing Huh-7 cells. *I,* DHRS3 immunofluorescence and BODIPY staining were performed after Huh-7 cells were treated with 100 μM ATO (n = 3). *J,* MTT assay detected the effect of ATO on DHRS3-mediated proliferation in Huh-7 cells (n = 3). *K* and *L,* Western blotting analysis of DHRS3 levels in Huh-7 (*L*) and HepG2 (*M*) cells treated with ATRA. *M* and *N,* the inhibitory effect of ATRA and ATO on the viability of Huh-7 (*N*) or HepG2 (*O*) cells was detected by MTT. The ZIP index calculates the synergistic effect of ATRA and ATO (ZIP > 0, synergistic effect). *O,* MTT assay detected the effect of DHRS3 knockdown on the inhibitory actions of ATRA, ATO, and their combination in Huh-7 cells (n = 3). All experiments were performed with “n” independent biological replicates. The data are presented as mean ± SD values. Unpaired *t-*tests (*A*–*F*) or one-way ANOVA (J and O) were used for statistical analysis. n.s.: not significant, ∗∗*p* < 0.01, ∗∗∗*p* < 0.001. DHRS3, short-chain dehydrogenase/reductase; ATO, atorvastatin; ATRA, all-trans retinoic acid; HCC, hepatocellular carcinoma; IHC, immunohistochemistry; MTT, 3-(4,5-dimethylthiazol-2-yl)-2,5-diphenyltetrazolium bromide.

To investigate the functional effect of DHRS3 on HCC cell proliferation, we performed 3-(4,5-dimethylthiazol-2-yl)-2,5-diphenyltetrazolium bromide (MTT) and colony formation assays in DHRS3-overexpressing Huh-7 cells. These experiments revealed that DHRS3 overexpression significantly enhanced Huh-7 or HepG2 cell clonogenic capacity (Fig. 6, *A* and *B*). Building on prior enrichment analyses suggesting DHRS3 involvement in cholesterol-related metabolism (Fig. 2, *D* and *E*). DHRS3 overexpression increased the total cholesterol and lipid droplet content in HCC cells (Fig. 6, *C*–*F*). Conversely, DHRS3 knockdown suppressed lipid droplet content in HCC cells (Fig. S4). The observation of IHC results of clinical samples in the preliminary experiment found that DHRS3 was expressed in the lipid droplet region of cancer cells in some samples (Fig. 6*G*). We conducted BODIPY staining coupled with immunofluorescence to localize DHRS3 subcellular distribution. DHRS3 predominantly localized to lipid droplets in Huh-7 cells (Fig. 6*H*). DHRS3 overexpression led to an increase in its content within lipid droplets and other cellular structures (Fig. 6*H*).

Notably, Inhibition of cholesterol synthesis by using ATO changed DHRS3 subcellular localization and reversed DHRS3-mediated proliferative enhancement without affecting baseline viability (Fig. 6*J*), indicating that DHRS3's oncogenic function is dependent on cholesterol metabolism. Given that ATRA upregulates DHRS3 expression (20, 21), we evaluated the therapeutic synergy between ATO and ATRA. Western blotting analysis confirmed that ATRA treatment significantly increased DHRS3 levels in HCC cells (Fig. 6, *K* and *L*). Combined treatment of ATO and ATRA synergistically inhibited HCC cell viability (Fig. 6, *M* and *N*). Furthermore, DHRS3 knockdown enhanced the inhibitory effect of ATRA on HCC cell viability while abolishing the sensitizing effect of ATO on ATRA action (Fig. 6*O*). These findings establish that DHRS3 promotes HCC cell proliferation, which was associated with cholesterol-related metabolism.

### ATO synergizes with ATRA to regulate DHRS3, promote antitumor immune cell infiltration, and inhibit HCC growth in vivo

**Figure 7:**
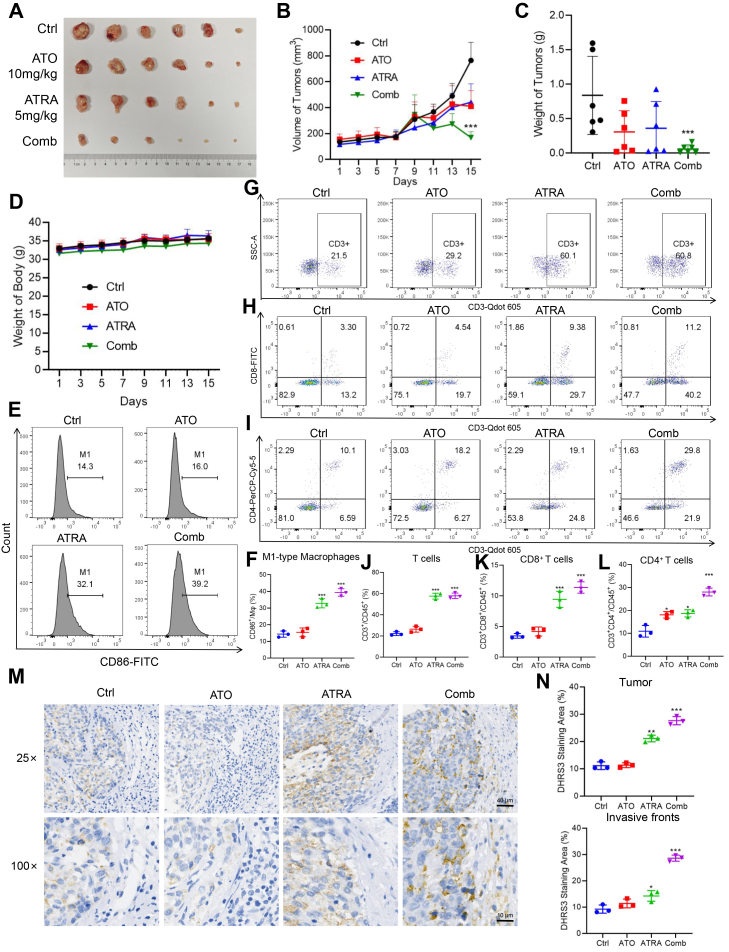
**ATO synergizes with ATRA to regulate DHRS3, promote antitumor immune cell infiltration, and inhibit HCC growth *in vivo*.***A,* mice bearing HCC xenografts were treated with ATO, ATRA, or combination. (n = 6 mice per group). *B,* changes in tumor volume (n = 6 mice per group). *C,* tumor weight (n = 6 mice per group). *D,* changes in mouse body weight (n = 6 mice per group). *E* and *F,* flow cytometry analysis of M1 macrophage infiltration in tumor tissues and statistical results (n = 3 mice per group). *G–L,* flow cytometry analysis of T cell, CD8+ T cell, and CD4+ T cell infiltration in tumor tissues (*G*–*I*) and statistical results (*J*–*L*) (n = 3 mice per group). *M,* IHC staining showing DHRS3 protein expression in tumor tissues (n = 3 mice per group). *N,* statistical analysis of DHRS3-positive areas in the tumor interior and invasive front (n = 3 mice per group). The data are presented as mean ± SD values. One-way ANOVA was used for statistical analysis (*B*, *C*, *D*, *F*, *J*, *K*, *L*, and *N*). ∗*p* < 0.05, ∗∗*p* < 0.01, and ∗∗∗*p* < 0.001. DHRS3, short-chain dehydrogenase/reductase; ATO, atorvastatin; ATRA, all-trans retinoic acid; HCC, hepatocellular carcinoma; immunohistochemistry.

To investigate the synergistic effect of ATO and ATRA on HCC *in vivo*, an ectopic H22 xenograft mouse model was performed. The subcutaneous implantation model offers technical simplicity, a high tumor take rate, and facilitates straightforward monitoring of tumor growth and volume measurement, which are advantageous for preliminary drug screening and efficacy assessment. Previous studies have demonstrated that either ATO (43) or ATRA (44) can inhibit the growth of HCC *in vivo* to a certain extent. Macroscopic observation of the excised tumors (Fig. 7*A*) showed that HCC tumors treated with ATO (10 mg/kg), ATRA (5 mg/kg), or the combination therapy were all smaller than those in the control group. Quantitative analysis of tumor volume (Fig. 7*B*) and weight (Fig. 7*C*) revealed that monotherapy of either ATO or ATRA and combination group inhibited HCC growth, while the combination group exhibited more pronounced reduction in HCC volume from day 9. Measurement of mouse body weight during the administration period showed no significant difference among the groups, indicating that the combination of the two drugs had no obvious systemic toxicity (Fig. 7*D*).

Further, we detected the infiltration of key antitumor immune cells within the tumors by flow cytometry. The results showed that ATRA treatment significantly increased the proportions of M1-type macrophages (Fig. 7, *E* and *F*), T cells (Fig. 7, *G* and *J*), and CD8^+^ T cells (Fig. 7, *H* and *K*) in the tumor tissues. Both ATO and ATRA alone increased the proportion of CD4^+^ T cells to a certain extent, while the combination of the two drugs further enhanced this proportion (Fig. 7, *I* and *L)*. IHC staining revealed results consistent with the previous spatial transcriptome analysis, DHRS3 was highly expressed in hepatocellular carcinoma cells but lowly expressed at the tumor margin (Fig. 7*M*). ATRA treatment significantly increased the level of DHRS3 in tumor tissues, while the combination of the two drugs further elevated the DHRS3 level at the tumor margin (Fig. 7, *M* and *N*).

In conclusion, these results demonstrate that ATO synergizes with ATRA to regulate DHRS3, promote antitumor immune cell infiltration, and inhibit HCC growth *in vivo*.

### Integration of DHRS3 with cholesterol metabolic signature predicts HCC prognosis and therapeutic sensitivity

**Figure 8:**
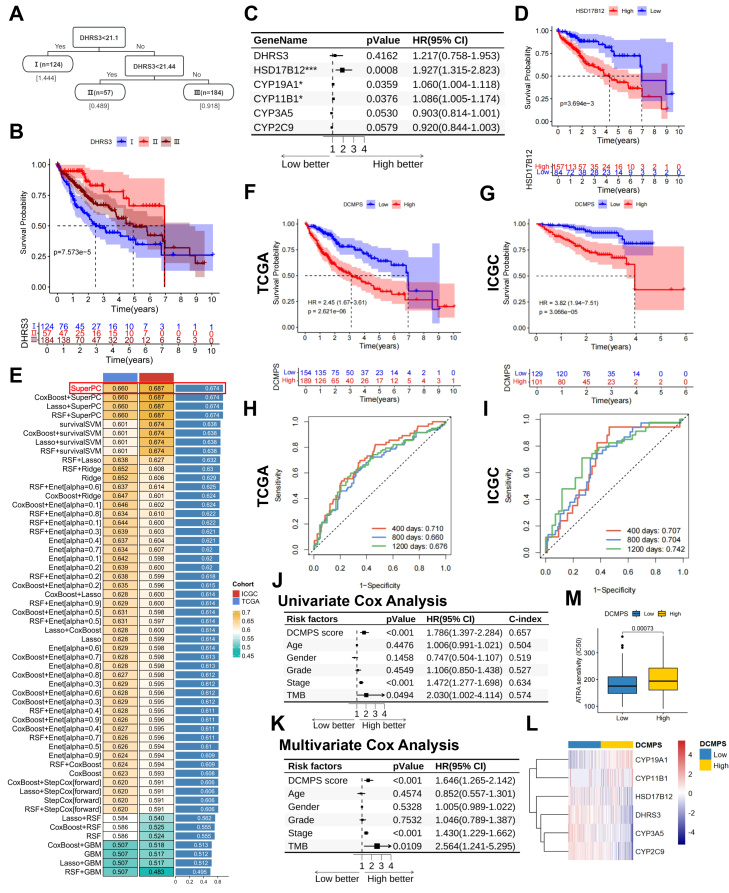
**DHRS3-cholesterol metabolic prognostic signature (DCMPS) enables prognostic stratification and therapeutic guidance in hepatocellular carcinoma.***A,* decision tree analysis of DHRS3 expression in relation to HCC patient prognosis. Cumulative hazard scores (higher values indicate poorer prognosis) are shown for distinct subgroups. *B,* Kaplan–Meier survival curves stratified by DHRS3 expression levels (group I: low DHRS3; group II: moderate DHRS3; group III: high DHRS3). *C,* univariate Cox regression analysis of DHRS3 and 49 cholesterol-related genes in the DHRS3-high subgroup. *D,* survival analysis of HSD17B12 expression in DHRS3-high patients. *E,* performance evaluation of 55 machine learning models using C-index in training (TCGA-LIHC) and validation (ICGC-LIRI-JP) cohorts. *F* and *G,* Kaplan–Meier curves demonstrating overall survival (OS) differences between high- and low-DCMPS groups in TCGA (*F*) and ICGC (*G*) cohorts. *H* and *I,* time-dependent ROC curves evaluating DCMPS prognostic accuracy at 400, 800, and 1200 days in TCGA (H) and ICGC (I) cohorts. *J* and *K,* univariate (*J*) and multivariate (*K*) Cox regression analyses of prognostic factors. *L,* heat map showing DCMPS risk scores and expression patterns of signature genes. *M,* predicted ATRA sensitivity (IC50 values) in high- *versus* low-DCMPS groups. One-way ANOVA (*M*) and log-rank tests (*B*, *D*, *F*, and *G*) or Cox proportional hazard regression models (*C*, *J*, and *K*) were used for statistical analysis. DHRS3, short-chain dehydrogenase/reductase; C-index, concordance index; TCGA, The Cancer Genome Atlas; LIHC, liver hepatocellular carcinoma; DCMPS, DHRS3-cholesterol metabolic prognostic signature.

To determine whether the detection of DHRS3 combined with cholesterol metabolism-related genes could improve prognostic accuracy in HCC, we first employed a decision tree algorithm to dissect the relationship between DHRS3 expression and patient outcomes (Fig. 8*A*). The cumulative hazard score, reflecting mortality risk, was highest in group I (DHRS3-low), indicating the poorest prognosis. Intriguingly, groups II and III (DHRS3-high) exhibited divergent survival patterns, with excessively high DHRS3 expression paradoxically correlating with adverse outcomes (Fig. 8*B*). This phenomenon may result from excessive DHRS3 expression driving HCC proliferation via cholesterol metabolic reprogramming. Consequently, prognosis assessment based solely on DHRS3 levels proves inadequate, necessitating novel biomarkers to stratify DHRS3 high patients.

Univariate Cox analysis of DHRS3 and 49 cholesterol metabolism-related genes in the DHRS3-high subgroup identified HSD17B12 (HR = 1.927 (95% confidence interval [CI]: 1.315−2.823), *p* < 0.001) as the strongest prognostic predictor, while DHRS3 itself showed no independent prognostic value (Fig. 8*C*). HSD17B12, a critical 17β-hydroxysteroid dehydrogenase, mediates fatty acid elongation (45) and orchestrates large lipid droplet biogenesis (46). Survival analysis confirmed that HSD17B12 effectively stratified prognosis in DHRS3-high patients (Fig. 8*D*).

Using a LOOCV framework, we integrated DHRS3 with five cholesterol-related genes (HSD17B12, CYP19A1, CYP11B1, CYP3A5, CYP2C9) to construct 55 machine learning models. The supervised principal components analysis algorithm demonstrated superior performance in both TCGA-LIHC (training concordance index [C-index] = 0.660) and ICGC-LIRI-JP (validation C-index = 0.687) cohorts (Fig. 8*E*). Patients stratified by the DCMPS showed markedly divergent survival, with high-DCMPS groups exhibiting significantly reduced OS in TCGA (HR = 2.45 (95% CI: 1.67−3.61), *p* < 0.001) (Fig. 8*F*) and ICGC datasets (HR = 3.82 (95% CI: 1.94−7.51), *p* < 0.001) (Fig. 8*G*).

Time-dependent ROC analysis found robust prognostic discrimination, with area under the curves of 0.710 (400 days), 0.660 (800 days), and 0.676 (1200 days) in TCGA (Fig. 8*H*), and 0.707, 0.704, and 0.742, respectively, in ICGC (Fig. 8*I*). Univariate Cox regression analysis identified the DCMPS score (HR = 1.786, 95% CI: 1.397–2.284, *p* < 0.001; C-index = 0.657), TNM stage (HR = 1.472, 95% CI: 1.277–1.698, *p* < 0.001; C-index = 0.634), and TMB score (HR = 2.030, 95% CI: 1.002–4.114, *p* = 0.0494; C-index = 0.574) as significant predictors of adverse prognosis (Fig. 8*J*). Notably, the DCMPS score demonstrated superior prognostic accuracy compared with other clinicopathological variables, as evidenced by its higher C-index value. Crucially, multivariate analysis confirmed the DCMPS score as an independent prognostic factor for hepatocellular carcinoma (LIHC) patients (HR = 1.646, 95% CI: 1.265–2.142, *p* < 0.001) after adjusting for confounders (Fig. 8*K*).

The high-risk DCMPS subgroup exhibited DHRS3 downregulation with concomitant upregulation of steroidogenic enzymes CYP19A1 and CYP11B1 (Fig. 8*L*). Drug sensitivity prediction *via* oncoPredict revealed significantly higher ATRA IC50 values in high-risk patients (Fig. 8*M*), suggesting that reduced ATRA responsiveness could be reversed by cholesterol synthesis inhibitors like ATO.

Collectively, the DCMPS model, constructed by integrating DHRS3 with cholesterol metabolism signatures, not only achieves precise prognostic stratification in HCC but also guides the therapeutic application of ATRA and ATO in HCC.

## Discussion

**Figure 9:**
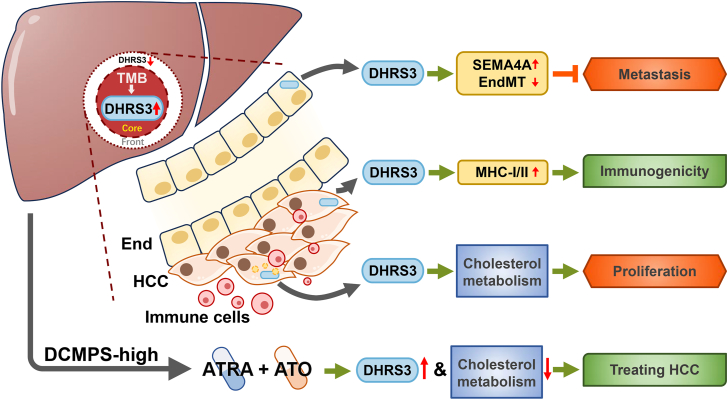
**Schematic illustration of the dual regulatory mechanisms of DHRS3 in hepatocellular carcinoma (HCC).** In hepatocytes, DHRS3 promotes cholesterol metabolism-driven tumor proliferation, which can be targeted by atorvastatin (ATO). Concurrently, DHRS3 enhances MHC-I/II-mediated antigen presentation, activating antitumor immunity. In endothelial cells, DHRS3 reinforces SEMA4A signaling and suppresses endothelial-mesenchymal transition (EndMT), thereby inhibiting metastasis. The combination of ATO and all-trans retinoic acid (ATRA) synergistically suppresses tumor growth by concurrently blocking DHRS3-mediated cholesterol metabolism and augmenting immune activation. The DHRS3-cholesterol metabolic prognostic signature (DCMPS) enables risk stratification and guides therapeutic strategy. DHRS3, short-chain dehydrogenase/reductase.

This study first reveals the tissue-specific association between DHRS3 expression and prognosis through pan-cancer analysis. Notably, elevated DHRS3 expression in tumor *versus* normal tissues persists as a favorable prognostic indicator in four malignancies including LIHC. Tumor DHRS3 levels demonstrate a positive correlation with TMB, suggesting its compensatory tumor suppressor upregulation during malignant progression. Single-cell and spatial transcriptomic analyses identify DHRS3-specific enrichment in malignant hepatocytes and endothelial cells within tumor core regions, contrasting with marked downregulation at invasive front areas. Mechanistically, DHRS3 enhances MHC class I/II antigen presentation in hepatocytes while suppressing metastasis-associated EndMT in tumor vasculature, the latter process involving SEMA4A signaling. This dual mechanism establishes enhanced immune surveillance in DHRS3-high tumors, consistent with established immune-regulatory functions of SEMA proteins (47) and MHC-mediated antigen presentation (48). This evidence provides mechanistic justification for DHRS3 upregulation in radiation-induced thyroid cancer models (25) and its inverse correlation with metastatic potential (24). DHRS3-low cells form a protective barrier against immune attack at tumor periphery while acquiring metastatic competence through differentiation programming (Fig. 9).

An unexpected finding revealed that DHRS3 overexpression promotes HCC proliferation, uncovering a previously unrecognized role of this gene in metabolic regulation. Lipid droplet localization assays and cholesterol metabolism inhibition experiments demonstrated that its pro-proliferative effect is dependent on cholesterol metabolic pathways under its regulation. This aligns with recent findings demonstrating retinol-metabolizing enzymes' involvement in lipid homeostasis regulation (49). The discovery suggests a potential link between DHRS3's previously observed oncogenic role in triple-negative breast cancer (23) and the lipid metabolic reprogramming characteristic of this malignancy. Although DHRS3 exhibits metabolism-dependent pro-proliferative effects, its tumor-promoting activity remains strictly context-dependent. Lipid droplet localization assays revealed that DHRS3 exerts proliferative effects exclusively in cholesterol metabolically active tumor cells, with complete abrogation observed upon ATO intervention. This microenvironmental specificity in metabolic regulation implies that DHRS3's oncogenic function may operate as a compensatory mechanism for immune activation. Clinical data analyses further substantiate this notion, showing worsened prognosis in DHRS3-overexpressing patients only when accompanied by cholesterol metabolism abnormalities, whereas isolated DHRS3 elevation consistently correlates with favorable outcomes. However, it is important to note that our total cholesterol measurements do not distinguish between free and esterified cholesterol pools. Therefore, the specific cholesterol-derived metabolites or the exact metabolic pathways involved require further investigation.

Notably, the cholesterol synthesis inhibitor ATO not only reverses DHRS3-mediated pro-proliferative effects but also synergizes with ATRA to exert enhanced tumor suppression. Conventional ATRA monotherapy may inadvertently induce compensatory protumor effects through DHRS3 upregulation, whereas combining ATO overcomes this therapeutic limitation. *In vivo* studies in the xenograft mouse model demonstrated that the coadministration of ATO and ATRA significantly inhibited HCC tumor growth and promoted the infiltration of antitumor immune cells. Mechanistically, ATRA promotes immune-mediated tumor control through DHRS3 upregulation, whereas ATO eliminates DHRS3-driven cholesterol-dependent proliferation, establishing a dual-axis therapeutic strategy for HCC.

The machine learning–based DCMPS model, integrating DHRS3 expression with cholesterol metabolism signatures, demonstrates significantly superior prognostic predictive performance than traditional TNM staging. This advantage likely stems from DCMPS's capacity to capture the dynamic equilibrium between tumor immune microenvironment activation and metabolic reprogramming states—dimensions overlooked by anatomically focused TNM staging. Furthermore, the DCMPS score is associated with ATRA sensitivity in retrospective analyses, providing a rationale and a potential biomarker framework for future studies aimed at evaluating ATO-coordinated therapies.

This study systematically elucidates the dual regulatory mechanisms of DHRS3 in HCC tumor-suppressive effects *via* immune activation within the tumor microenvironment and pro-proliferative effects through cholesterol metabolism at the cellular level. The developed DCMPS model integrates prognostic stratification with therapeutic sensitivity prediction, offering a novel tool for HCC precision medicine. However, limitations warrant attention: (1) the retrospective study design may introduce selection bias, necessitating validation in prospective cohorts to confirm DCMPS clinical applicability; (2) the mechanisms by which mutations drive DHRS3 upregulation, and the specific molecular pathways through which DHRS3 enhances antigen presentation to activate antitumor immunity, await further investigation; (3) the biological functions of cholesterol metabolism-related genes within the DCMPS model, their synergy with DHRS3, and the potential involvement of cholesterol-derived metabolites (e.g., oxysterols) and their associated signaling pathways remain to be elucidated; and (4) the precise molecular mechanisms underlying the observed synergistic effect between ATRA and ATO, particularly how they functionally interplay through or alongside DHRS3, await deeper exploration. Future work will establish patient-derived xenograft models and organoid platforms to dissect DHRS3's dual mechanisms and explore synergistic therapeutic strategies combining DHRS3-targeted interventions with immune/metabolic modulators.

This study demonstrates that DHRS3, a key enzyme in retinol metabolism, is significantly upregulated in HCC. Elevated DHRS3 expression is associated with a favourable patient prognosis. Functionally, DHRS3 remodels the antitumor microenvironment by enhancing antigen presentation *via* MHC-I/II and suppressing prometastatic endothelial traits through the EndMT and SEMA4A pathway, although it also promotes tumor proliferation *via* cholesterol metabolic reprogramming. The DCMPS surpasses conventional staging systems in predicting patient outcomes and provides a rationale for combination therapy with ATRA and ATO, which synergistically inhibits tumour growth and counteracts therapeutic resistance. These findings elucidate the immunometabolic role of DHRS3 in HCC and offer a biomarker-guided strategy for precision therapy in liver cancer.

## Experimental procedures

### Tissue microarray ethics statement

The human tissue study was conducted in accordance with the principles of the Declaration of Helsinki. The study using the tissue microarray was approved by the Life Sciences Ethics Committee of Hunan Aifang Biotechnology Co., Ltd. The query code is HN20250401. The specific clinical characteristics of the patients are shown in Table S1.

### Mendelian randomization analysis

Genetic instruments for DHRS3 expression (ENSG00000162496) were obtained from the eQTLGen Consortium (50), which comprises large-scale eQTL summary data derived from blood-derived samples, predominantly of European ancestry. SNPs significantly associated with DHRS3 expression (*p* < 5 × 10^−8^) were selected as instrumental variables. Summary-level genome-wide association study (GWAS) data for the outcome, “malignant neoplasm of connective and soft tissue,” were sourced from publicly available GWAS repositories integrated within the DMRdb framework (51). This database provides curated and standardized GWAS datasets that have undergone rigorous quality control procedures. The two-sample Mendelian randomization approach was employed to estimate the causal effect of genetically predicted DHRS3 expression on the risk of connective and soft tissue malignancy. All statistical analyses were conducted using R software (https://www.r-project.org/) with the TwoSampleMR package, aligning with the standardized pipeline implemented in the DMRdb resource.

### Spatial transcriptomics analysis

The analysis focused on an invasive front sample from a HCC case HCC-1L (35), obtained from a 54-year-old male patient (stage II, T2N0M0). Spatial transcriptomics analysis was performed using the SPATA2 package (version 3.1.0) in R v4.2.1. The raw spatially resolved gene expression data and associated spatial coordinates were imported and processed within the SPATA2 framework to create a spatially aware data object.

### Cell culture

Human HCC cell line Huh-7 and HepG2, liver endothelial cell line SK-HEP-1, and monocyte cell line THP-1 were obtained from the National Collection of Authenticated Cell Cultures. Huh-7, HepG2, and SK-HEP-1 cells were cultured in Dulbecco's modified Eagle's medium/F-12 medium (Gibco), and THP-1 cells was cultured in RPMI 1640 medium (Cienry, Huzhou) supplemented with 10% fetal bovine serum (Sijiqing) at 37 °C in 5% CO_2_. The culture medium was replaced every other day, and cells were passaged every 2 days.

### Colony formation assay

The cells were digested and collected, and inoculated into 24-well plates at 1 × 10^3^ cells per well. After being placed in a 37 °C, 5% CO_2_ incubator for 14 days, the cells were fixed using 4% paraformaldehyde for 25 min, and then stained with 0.1% crystal violet staining solution. ImageJ (version 1.54, https://imagej.net/ij/) software was used to calculate the number of colonies.

### MTT assay

The cells collected as above were inoculated into 96-well plates (4 × 10^3^ cells per well). After being placed in a 37 °C, 5% CO_2_ incubator for 48 h, 20 μl of MTT reagent (5 mg/ml) was added to each well and incubation was continued for 4 h. Subsequently, formazan crystals were completely dissolved using dimethyl sulfoxide, the absorbance at 490 nm of each well was measured by microplate reader (Bio-Rad).

### Wound healing assay

The cells collected as above were inoculated into 24-well plates at 2 × 10^5^ cells per well and cultured to confluence. Scratches were scraped in monolayer cells and recordings were taken by microscope (LH-M100CB-1, Nikon) at 0 and 12 h to assess the migration level.

### Reverse transcription and quantitative real-time PCR

The total RNA in the samples was extracted using Trizol and complementary DNA was synthesized using the Evo M-MLV reverse transcription kit (Accurate, Hunan). Real-time quantitative PCR was subsequently performed using the SYBR Green Pro Taq HS premixed qPCR kit (Accurate, Hunan). The relative RNA expression was determined by the 2^−ΔΔCt^ method. PCR primer sequences were listed as follows:

SEMA4A forward: 5′-AGCTCCCCACATCTACGCA-3′;

reverse: 5′-AGAAGGCACAAACCGCAGAG-3'; HLA-DMA forward: 5′-CCTGCACACAGTGTACTGC-3′;

reverse: 5′-CACCCGAGTGTTCTGGGAA-3'; HLA-G forward: 5′-GAGGAGACACGGAACACCAAG-3′;

reverse: 5′-GTCGCAGCCAATCATCCACT -3'; DHRS3 forward: 5′-ACTGAGTGCCATTACTTCATCTG-3′;

reverse: 5′-CATCACTGTCCATTAGGCTCTTC-3'; GAPDH forward: 5′-GGAGCGAGATCCCTCCAAAAT-3′;

reverse: 5′-GGCTGTTGTCATACTTCTCATGG-3′.

### Western blotting

Cells were collected and lysed by radio-immunoprecipitation assay lysate–containing phosphatase inhibitor and protease inhibitor. Equal masses of proteins were separated by SDS-PAGE gel electrophoresis and transferred to polyvinylidene fluoride membranes. After incubation with 5% nonfat milk, membranes were incubated with primary antibodies and further incubated with horseradish peroxidase-conjugated secondary antibody. Protein complexes were detected by ECL (Clinx). Primary antibodies in the study were antibodies against DHRS3 (1:1000, abcam, #1018196-8), CD31 (1:1000, abclonal, #A19014), α-SMA (1:1000, abclonal, #A17910), Vimentin (1:1000, abcam, #2862-1), GAPDH (1:1000, Cst, #5174).

### Determination of total cholesterol content

To measure the intracellular total cholesterol content, Huh-7 or HepG2 cells subjected to respective treatments were harvested. After washing twice with ice-cold PBS, the cells were lysed on ice for 30 min using a cell lysis buffer. The lysates were then centrifuged at 12,000*g* for 10 min at 4 °C, and the resulting supernatants were collected as samples for analysis. A commercial Total Cholesterol Assay Kit (NJJCBIO, A111-1-1) was used according to the manufacturer's instructions. Briefly, samples, standards, and working reagent were added sequentially into a 96-well plate. After thorough mixing, the plate was incubated at 37 °C in the dark for 30 min. The absorbance of each well was measured at 500 nm using a microplate reader (Bio-Rad,). The cholesterol concentration in each sample was calculated based on a standard curve and normalized to the total protein concentration determined by the bicinchoninic acid assay. The final results are expressed as micrograms of cholesterol per milligram of protein (μg/mg protein).

### Development and validation of the DCMPS through integrative machine learning approaches

Two bulk-seq cohorts were downloaded to examine the robustness and clinical value of DCMPS model. Those included 349 individuals from TCGA (https://portal.gdc.cancer.gov/) TCGA-LIHC cohort, 196 individuals from the International Cancer Genome Consortium (https://dcc.icgc.org/) ICGC LIRI-JP cohort.

Two independent datasets were utilized with a strict separation between training and external validation. The TCGA-LIHC cohort served exclusively as the training set for model development, including feature selection and parameter tuning. The ICGC LIRI-JP cohort was held out and used strictly as an independent external test set to evaluate the final model's performance. The cohorts were processed and analyzed separately; they were not merged at any stage prior to the final validation step. The following integrative algorithms were used: elastic net regression (Enet), Lasso regression, Ridge regression, stepwise Cox regression, random survival forest, supervised principal components analysis, Survival Support Vector Machine, and partial least squares regression for Cox models. Among these methods, ridge regression applies an L2 penalty to shrink coefficients toward zero without setting them exactly to zero, thus not performing feature selection. In contrast, Lasso regression employs an L1 penalty to reduce some coefficients to exactly zero, thereby enabling automatic feature selection. Enet combines both L1 and L2 penalties, controlled by the parameter α, to achieve a balance between feature selection and coefficient shrinkage. Notably, Enet, Lasso, and RSF all offer feature selection capabilities, with Elastic Net providing a flexible approach that adapts based on the value of α. Enet, stepwise Cox regression, and RSF possess feature selection capabilities, with Enet offering a flexible approach that adapts based on the value of α.

Algorithmic combinations were used to develop predictive models for prognostic outcomes. We computed Harrell's C-index for each dataset, and the optimal model was determined by selecting the one that exhibited the highest average C-index in the validation cohort. To optimize the algorithm, a five-fold cross-validation approach was employed to screen for optimal hyperparameters, under the following settings: ntree was varied from 1000 to 10,000 in increments of 1000; mtry was set to values of 1 through 5; and nodesize was configured at 3, 6, 9, 12, and 15. The combination yielding the best C-index was then selected for the final model.

Additionally, the R package “timeROC” was used to conduct time-dependent area under the curve evaluations for survival variables (52). To identify independent prognostic indicators, univariate and multivariate Cox regression analyses were performed using the R packages “survival” and “survminer.

### Functional enrichment analysis

To investigate the biological functions of DHRS3, ClueGO was employed for functional enrichment analysis (53). Utilizing the WebGestalt database (http://www.webgestalt.org/) and R package “clusterProfiler,” gene ontology annotation, and Kyoto Encyclopedia of Genes and Genomes pathway enrichment analyses were conducted (54). Differential analysis results were used to systematically discern differences between the groups. The Wilcoxon rank-sum test was used to calculate the disparities between two groups, and genes exhibiting a *p* < 0.01 and |log2FC| > 1 were selected for further analysis.

### Evaluation ATRA sensitivity in the high- and low-DCMPS groups

The R package “oncoPredict” was utilized to predict drug responses in patients with HCC (55). The training dataset was obtained from the Genomics of Drug Sensitivity in Cancer database (56). Differences in sensitivity to ATRA between patients in the high- and low-DCMPS groups were analyzed by using an unpaired *t* test. Statistical significance was defined as *p* < 0.05.

### The relationship between DHRS3 gene expression and TMB or MSI

TMB was assessed using the R package “maftools.” MSI was calculated using the Tumor Immune Dysfunction and Exclusion algorithm (57). Spearman's rank correlation analysis was performed to evaluate the correlation between DHRS3 expression and TMB or MSI.

### Preprocessing and analysis of scRNA-seq data

The scRNA-seq dataset (GSE125449 (58)) was processed using Seurat v4. Cells expressing 500 to 6000 genes and containing 1000 to 100,000 unique molecular identifiers were preselected based on expression profiles. Filtering criteria included a mitochondrial gene content of less than 15%. After filtering, the dataset was normalized with Seurat's SCTransform, and the top 3000 most variable genes (HVGs) were selected to stabilize unique molecular identifiers count variance.

Principal component analysis was performed using HVGs. A shared nearest neighbor graph and uniform manifold approximation and projection were constructed with the Louvain algorithm, utilizing the first 30 principal components for clustering. Major cell types were annotated using the R package SingleR.

To predict copy number alterations without tumor annotations, CopyKAT (59) was employed to identify aneuploid cell clusters. Cell-cell communication analysis was conducted with CellChat (60), and cell trajectory analysis was conducted with monocle2 (61).

### DRHS3 expression pattern and prognostic analysis in human pan-cancer

The UCSC Xena platform was used for accessing TCGA and GTEx databases pertaining to pan-cancer DRHS3 expression levels and corresponding clinical characteristics (62). Data from the TCGA and GTEx databases were merged to compare DRHS3 mRNA levels between tumors and normal tissues for 33 cancer types, including adrenocortical carcinoma, bladder urothelial carcinoma, breast invasive carcinoma, cervical squamous cell carcinoma, cholangiocarcinoma, colon adenocarcinoma, lymphoid neoplasm diffuse large B cell lymphoma, esophageal carcinoma, glioblastoma, brain lower grade glioma, head and neck squamous cell carcinoma, KICH, kidney renal clear cell carcinoma, kidney renal papillary cell carcinoma, acute myeloid leukemia, LIHC, lung adenocarcinoma, lung squamous cell carcinoma, mesothelioma, ovarian serous cystadenocarcinoma, pancreatic adenocarcinoma, pheochromocytoma and paraganglioma, prostate adenocarcinoma, rectum adenocarcinoma, SARC, SKCM, stomach adenocarcinoma, testicular germ cell tumors, THCA, thymoma, UCEC, uterine carcinosarcoma, and uveal melanoma. The R “ggplot2” package was used for analyses of differential gene expression. Boxplots were used to present differences in expression levels across cancer subtypes or stages.

The connection between the DHRS3 expression and the prognosis of patients, including OS in 33 types of cancer was examined using forest plots and Kaplan–Meier curves. The surv_cutpoint function from the survminer R package was employed to determine optimal cutoffs for DHRS3 high/low expression groups. The HRs and 95% CIs were calculated using univariate survival analysis.

### Preparation of plasmid expression vector and shRNA

DHRS3 (DHRS3 complementary DNA were cloned into the lentivirus vector GV513) were obtained from Vigene biosciences (Shandong). shRNAs for DHRS3 (DHRS3 shRNAs were cloned into the lentivirus vector PCLenti-U6-CMV-Puro-WPRE) were obtained from OBiO Technology. Four types of recombinant plasmids were transfected into cells with Lipofectamine 2000 (Invitrogen). The empty vector plasmid (OE-NC) was transfected to serve as the negative control for the overexpression experiments. The DHRS3 overexpression plasmid (OE-DHRS3) was transfected to upregulate DHRS3 expression. The scrambled shRNA plasmid (sh-NC) was transfected to serve as the negative control for the knockdown experiments. The DHRS3-shRNA plasmids (sh-DHRS3) were transfected to specifically knock down the expression of DHRS3. The target sequences of DHRS3-shRNA were listed as follows:

#1:5′-GCACAGGACTGATGGGTATAA-3′;
#2:5′-GTTCCCTCTACAGATGATCTA-3′;
#3:5′-ACCTGCATGAACACTTTCAAA-3′.

### Macrophage generation and differentiation

THP-1 monocytic cells (1 × 10^6^ cells) were differentiated into macrophages in 60 mm dishes containing 3 ml of the RPMI 1640 medium (Cienry, Huzhou) containing 50 ng/ml phorbol 12-myristate 13-acetate over 48 h. For the coculture, OE-NC or OE-DHRS3 Huh-7 cells and the THP-1–derived macrophages were seeded together in 6-well plates at a 1:1 ratio, with a total density of 2 × 10^5^ cells per well. After 48 h of culture, the cells were collected, centrifuged, washed with PBS, and resuspended. Macrophages were stained and labeled with F4/80 APC (BioLegend). Further, M1-type macrophages were labeled with CD86 FITC (Thermo Fisher Scientific). Then, the proportion of macrophage polarization was detected by flow cytometry (Minipore) to evaluate the effect of Huh-7 cells overexpressing DHRS3 on the polarization of cocultured macrophages. FlowJo 10.8.1 (https://www.flowjo.com/) was used to analyze and visualize data.

### Animal ethics statement

Male Institute of Cancer Research mice (aged 4–5 weeks, body weight 18 ± 2 g) were purchased from Hangzhou Qizhen Experimental Animal Technology Co., Ltd, with license number SCXK (Zhe) 2024-0038. Mice were housed in a temperature (22 ± 2 °C) and humidity (55 ± 5%) controlled SPF facilities in a 12 h light/dark cycle with *ad libitum* access to food and water for 7 days. All animal procedures were approved by the Institutional Animal Care and Use Committee of Zhejiang Chinese Medical University, with approval number: IACUC-20250207-01). This study was conducted in accordance with the recommendations of the Animal Care and Use Committee of School of Pharmaceutical Sciences in Zhejiang Chinese Medical University.

### Animal experiment

Institute of Cancer Research male mice were inoculated with 4 × 10^5^ H22 cells in the right axilla to establish an ectopic xenograft model of HCC *via* subcutaneous injection. A total of 24 model mice were randomly assigned to four groups using a random number table method, with six mice in each group: control group (administered 0.9% saline), ATO group (administered ATO at 10.0 mg/kg), ATRA group (administered ATRA at 5.0 mg/kg), and combination group (administered both ATO at 10.0 mg/kg and ATRA at 5.0 mg/kg). The dosages of ATO and ATRA were determined based on their respective clinical doses and the human-to-mouse body surface area conversion factor. Drug administration commenced on the second day after H22 cell inoculation, with daily intraperitoneal injections of ATO and/or ATRA administered at 24-h intervals over a continuous 14-day treatment period. The body weight of each mouse was recorded prior to each intraperitoneal injection.

#### Flow cytometry

For one batch of cultured cells, surface staining was performed as follows: LIVE/DEAD cell stains (Zombie Aqua Fixable Viability Kit, BioLegend) were added, along with anti-F4/80 and anti-CD86 antibodies for macrophages, and anti-CD3, anti-CD4, and anti-CD8 antibodies for T cells. The cells were then incubated for 30 min in the dark. After washing with PBS at 4 °C, flow cytometry was conducted to detect the F4/80^+^cell population and quantify the proportion of F4/80^+^CD86^+^M1-polarized macrophages. Additionally, the CD3^+^cell population was detected, and the proportions of CD3^+^CD4^+^and CD3^+^CD8^+^T cells were quantified.

#### Immunohistochemical

The protein expressions of DHRS3 (1:200, abcam, #1018196-8) in tumor tissues were detected following the instructions of the StreptAvidin–Biotin Complex IHC kit. Diaminobenzidine was used as the chromogenic substrate, and hematoxylin was applied for nuclear counterstaining. The immunoreactive proteins were visualized as yellowish-brown staining, and the staining results were quantitatively analyZed using ImageJ software.

### Statistical analysis

Data were shown as mean ± SD of at least three independent experiments. R v4.2.1 was used for all statistical analyses. Results were compared between groups with one-way ANOVA or Student's *t-tests*. Differences in DHRS3 expression among patients at different disease stages were assessed using the Kruskal–Wallis test. Kaplan–Meier curves and log-rank tests or Cox proportional hazard regression models were employed when conducting survival analyses. Spearman correlation coefficient values were used to evaluate relationships among variables. *p* < 0.05 was the cut-off threshold when defining significance (*∗p* < 0.05, *∗∗p* < 0.01, and *∗∗∗p* < 0.001; *n.s*.: not significant).

## Data availability

The authors declare that all relevant data of this study are available within the article or from the corresponding author on reasonable request. The public databases used were downloaded from The UCSC Xena platform (https://xena.ucsc.edu/) and the International Cancer Genome Consortium (https://dcc.icgc.org/). The raw data for Western blot can be found in the Supplementary Information (Fig. S4).

## Supporting information

This article contains supporting information.

## Conflict of interest

The authors declare that they have no conflicts of interests with the contents of this article.
