# Supplementary material for: The retinol-metabolizing enzyme DHRS3 coordinates antigen presentation, endothelial stability, and cholesterol metabolism to suppress hepatocellular carcinoma progression
Source: J Biol Chem. 2026 May 15;302(7):113168. doi: 10.1016/j.jbc.2026.113168 (PMC13264070; doi:10.1016/j.jbc.2026.113168)
Supplement: Supplementary Material [file mmc1.docx]

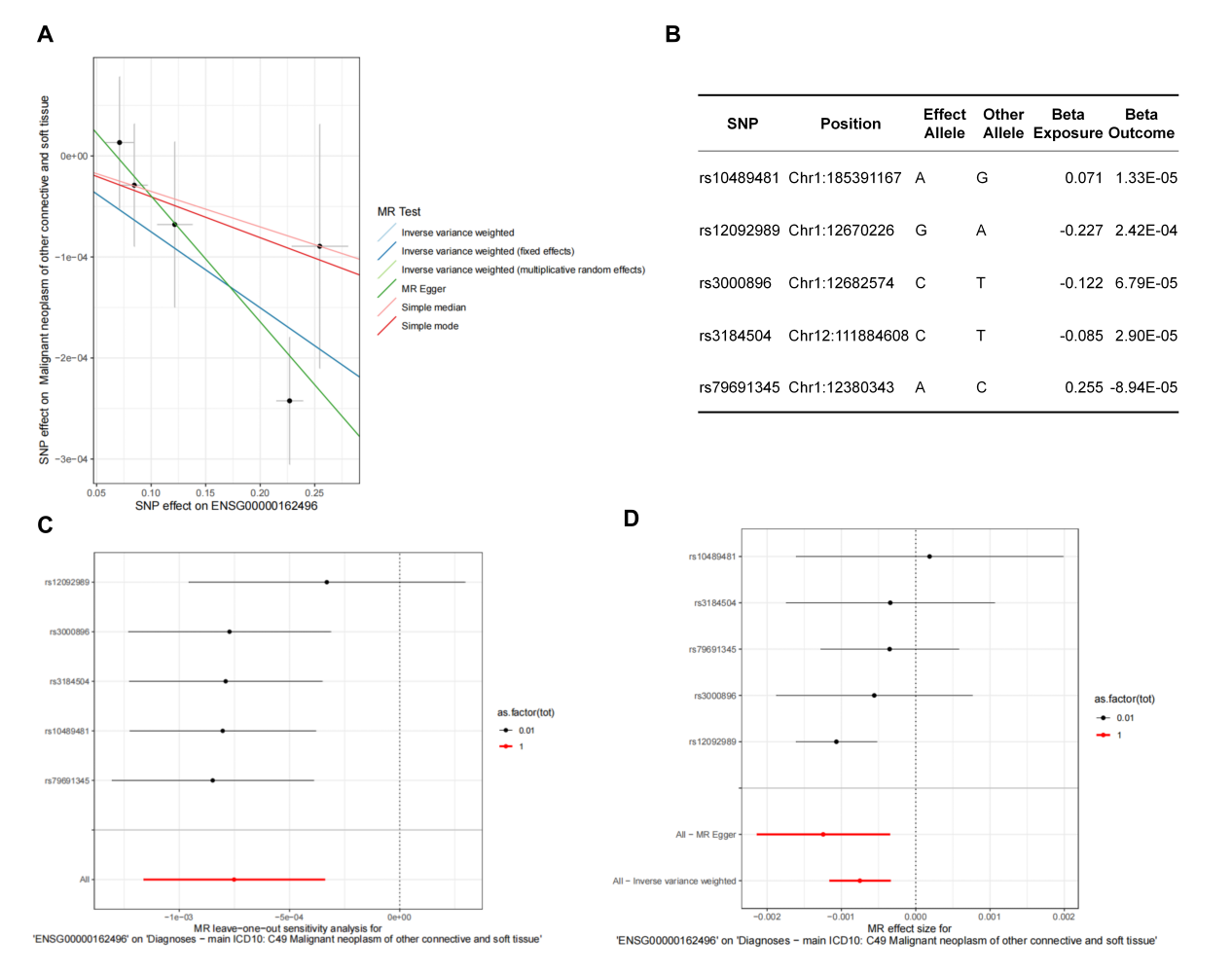


**Figure S1 Mendelian randomization study investigating the causal association between DHRS3 (ENSG00000162496) expression and Malignant neoplasm of connective and soft tissue.** A: Scatter plots showed the genetic associations of DHRS3 and Malignant neoplasm of connective and soft tissue. B: Detailed characteristics of SNPs. C: leave-one-out plots of DHRS3 and Malignant neoplasm of connective and soft tissue. D: Forest plots of SNPs associated with DHRS3 and Malignant neoplasm of connective and soft tissue.

**
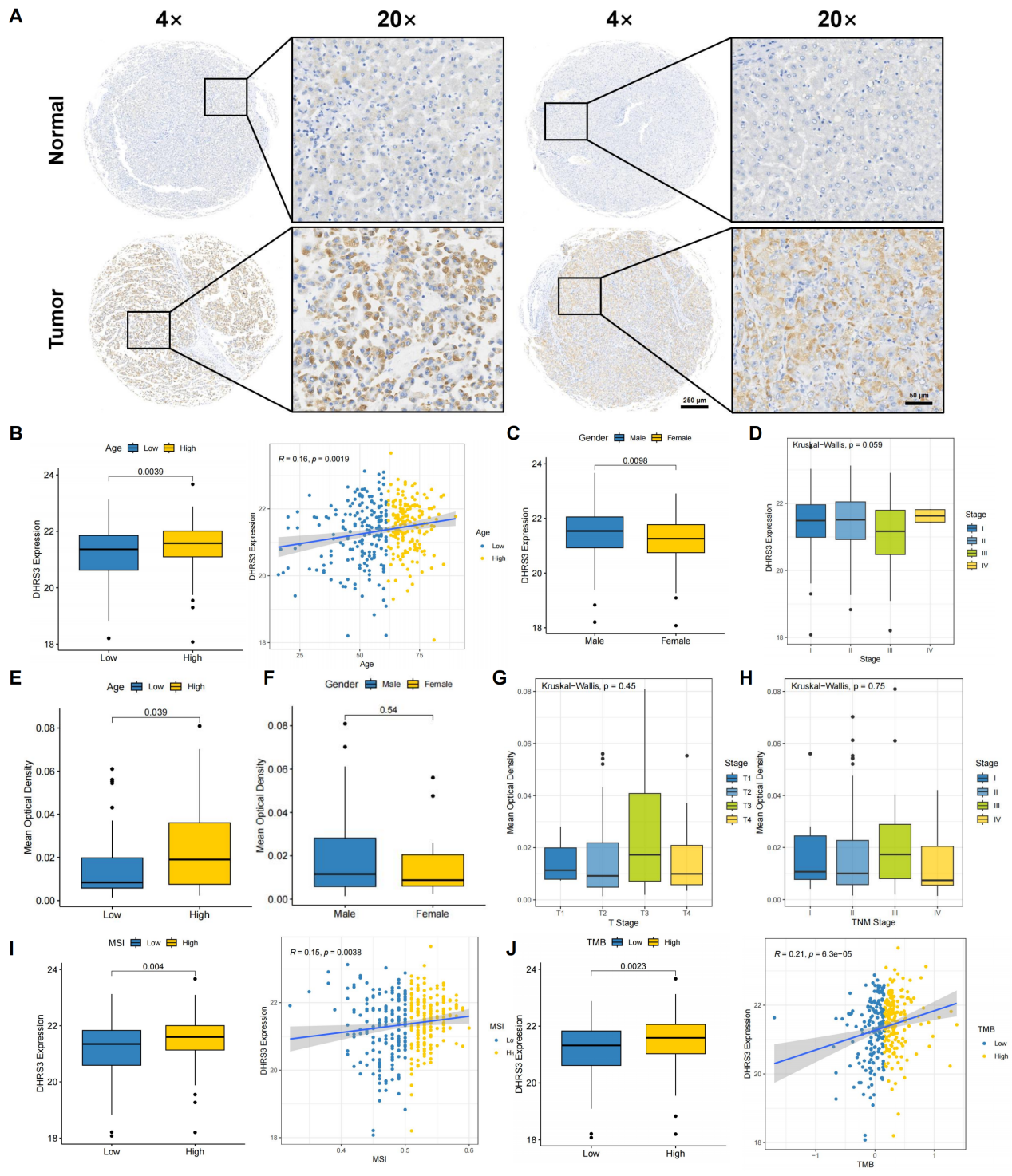
**

**Figure S2. Upregulated DHRS3 expression in HCC tissues with high tumor mutational burden (TMB) or microsatellite instability (MSI). (**A**) IHC analysis showed increased DHRS3 protein expression in HCC tissues compared with adjacent non-tumor tissues. (**B**) DHRS3 expression in TCGA-LIHC cohort (n=373) was positively correlated with patient age (left: DHRS3 expression differences between high/low age groups; right: Spearman correlation analysis). (**C**) Elevated DHRS3 expression in male HCC patients (TCGA-LIHC). (**D**) No association between DHRS3 expression and TNM staging in TCGA-LIHC cohort. (**E-H**) IHC validation in clinical HCC specimens confirmed DHRS3 upregulation in older patients (E), but no correlation with sex (F), TNM stage (G), or T stage (H). (**I,J**) DHRS3 expression in TCGA-LIHC cohort significantly correlated with MSI (I) and tumor TMB (J) (left: DHRS3 expression differences between high/low MSI/TMB groups; right: Spearman correlation analysis). One-way ANOVA test (B,C,E,F,I,J) or Kruskal-Wallis test (D,G,H)** were **used for statistical analysis..**


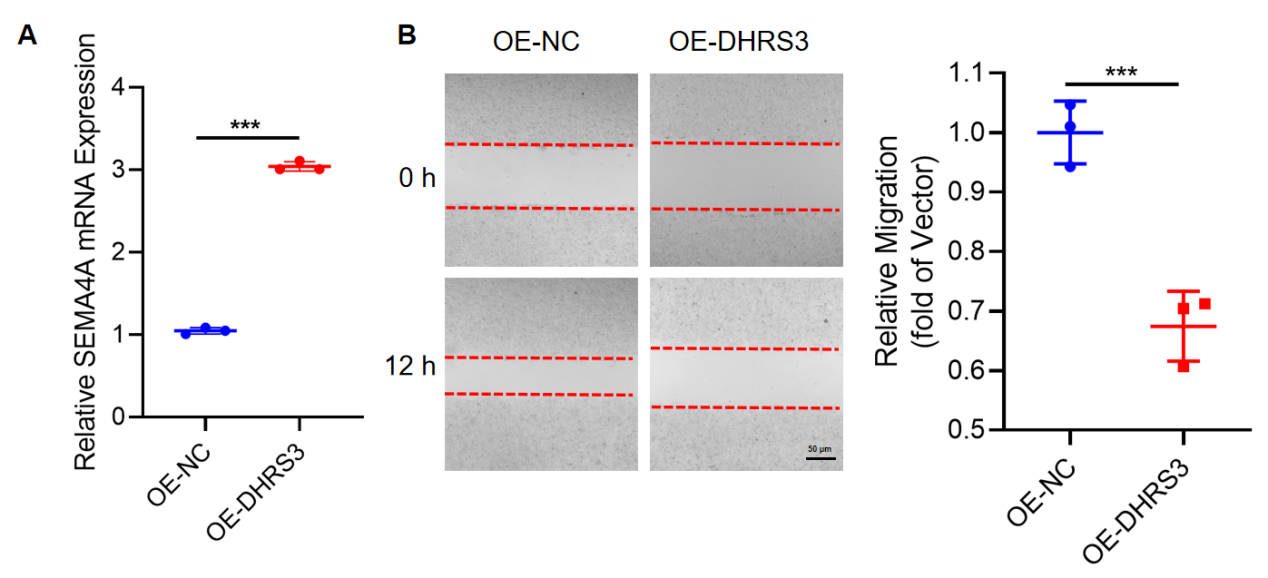


**Figure S3. DHRS3 overexpression enhances SEMA4A expression and inhibits endothelial cell migration. (A**) RT-qPCR analysis of SEMA4A mRNA levels in SK-HEP-1 cells after DHRS3 overexpression (n=3). (**B**) Wound healing assay quantifying migration capacity of DHRS3-overexpressing SK-HEP-1 cells (n=3). **All experiments were repeated three times. The data are presented as mean *± SD* values.** Student's t-tests **was used for statistical analysis. ****p* < 0.001.**


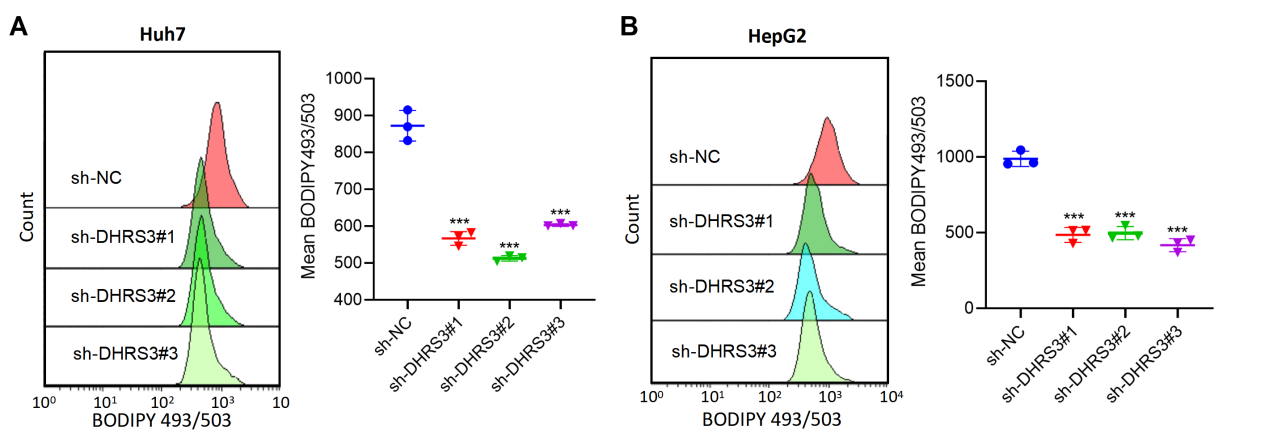


**Figure S4. DHRS3 knockdown reduces lipid droplet content in HCC lines. (**A, B**) BODIPY staining was used to detect the lipid droplet content in HCC lines Huh-7 (A) and HepG2 (B) upon DHRS3 knockdown (n=3).The data are presented as mean *± SD* values.** U**npaired** t-tests **was used for statistical analysis. ****p* < 0.001.**

**Table S1 The specific clinical characteristics of the patients**

| **Characteristics** |  |
| --- | --- |
| **Sex (No. (%))** |  |
| Female | 12(15) |
| Male | 68(85) |
| **Age (mean (SD))** | 54.28 (11.18) |
| **pT Stage (No. (%)) a** |  |
| T1 | 5(6.25) |
| T2 | 26(32.5) |
| T3 | 32(40) |
| T4 | 17(21.25) |
| **pN Stage (No. (%))b** |  |
| N0 | 77(96.25) |
| N1 | 2(2.5) |
| N2 | 1(1.25) |
| **pM Stage (No. (%))c** |  |
| M0 | 77(96.25) |
| M1 | 3(3.75) |
| **pTNM Stage (No. (%))d** |  |
| I/II | 55(68.75) |
| III/IV | 25(31.25) |
| **Follow-up Duration (mean (SD))** | 27.51 (18.73) |

（^a-d^ According to the American Joint Committee on Cancer/Union for International Cancer Control 8th staging system.）;
